# Professional Development of Nurse Anesthetists in China

**DOI:** 10.34172/ijhpm.9083

**Published:** 2025-10-22

**Authors:** Xi Zhang, Aimei Mao

**Affiliations:** ^1^Kiang Wu Nursing College of Macau, Macau SAR, China.; ^2^School of Nursing, Hunan Normal University, Changsha, China.

## Background

 Nurse anesthetists represent an evolutionary expansion of the nursing profession, assuming a crucial role in anesthesia care. The International Federation of Nurse Anesthetists (IFNA) defines nurse anesthetists as registered nurses who have fulfilled core anesthesia care education requirements and are formally credentialed and authorized to engage in anesthesia care practice within their respective national jurisdictions.^1^ “The China Health Statistics Yearbook 2022” reveals that the total number of surgeries performed in China amounted to 81.03 million in 2021, placing a significant burden on anesthesia care. In China, the terminology for nurse anesthetists has not yet been standardized, with terms such as “anesthesiology nurse,” “resuscitation room nurse,” and “anesthesia nurse” being used interchangeably. This paper adopts the term “nurse anesthetist.” It will provide an overview of challenges faced by nurse anesthetists in clinical practices and professional training. The contextual-based suggestions are also provided.

## The Slow but Steady Development of Anesthesia Care in China

 The development of anesthesia care in China has evolved gradually over the past few decades, driven by the growing demand for specialized care. While this article is not intended as a traditional bibliometric review, we incorporated selected bibliometric elements to illustrate publication trends and provide context for the development of nurse anesthesia research in China. Specifically, we examined publications indexed in three authoritative Chinese academic databases: China National Knowledge Infrastructure (CNKI), WanFang Data, and VIP Information Resource System from the beginning of relevant publications to the December 31, 2024. The search strategy incorporated the following subject terms: “anesthesia AND nursing” and “anesthesia AND nurse.” The search time frame spanned from the inception of each database to December 31, 2024, with the retrieval date being February 21, 2025. Literature related to anesthesia nursing theory and practice was included, while conference notifications, news reports, and other irrelevant studies were excluded. The retrieved literature was exported in RefWorks format, including titles, authors, keywords, institutions, etc. Duplicate entries were removed using EndNote 21 software, and we manually screened the remaining records to eliminate ineligible literature.

 The analysis revealed a consistent upward trend in the scholarly productivity of anesthesia nursing research in China by the year 2020 (Figure). There is a slight decrease in the number of publications during 2021–2024. It is important to note that some articles accepted in the later months of 2024 may not yet be publicly available.

**Figure:**
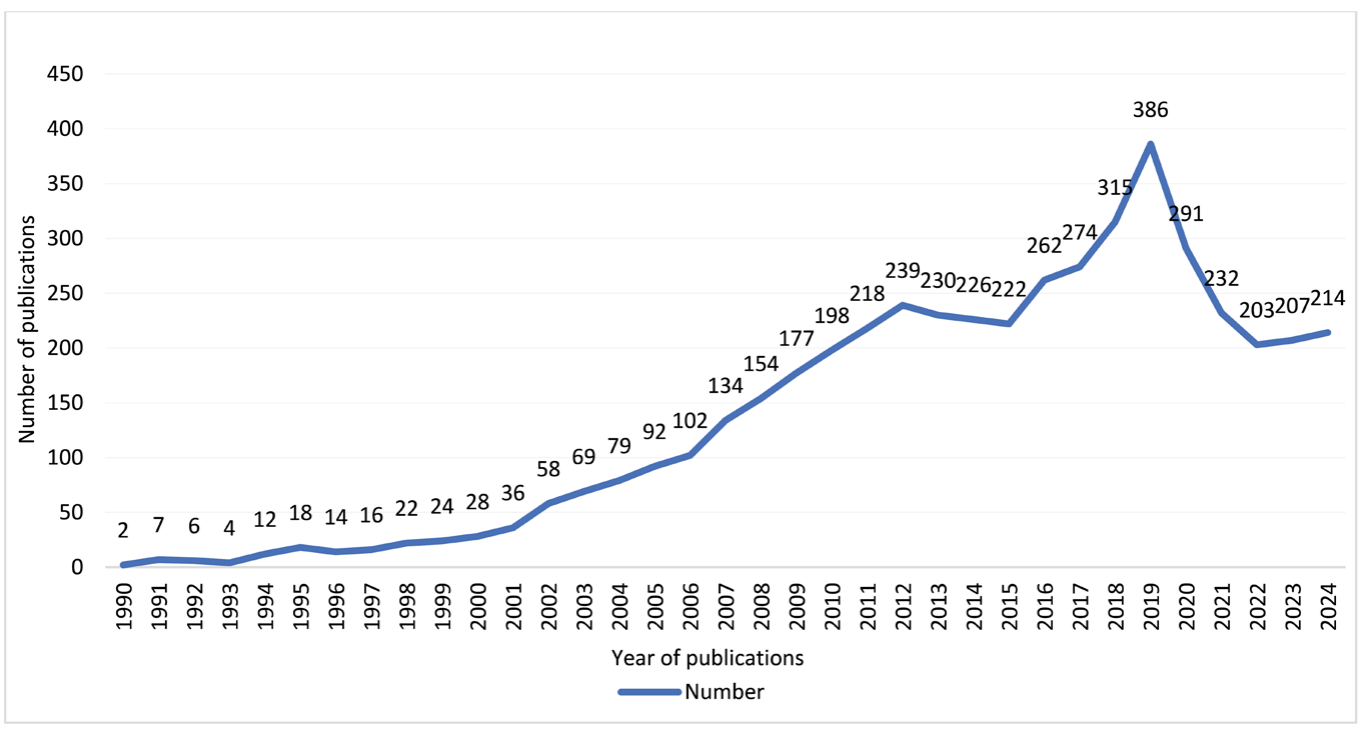
Trends in Anesthesia Nursing Research Publication in China.

 The path of the anesthesia care development in China can be divided into three stages:

1. Early stages (Pre-1990s). Anesthesia care in China was largely handled by anesthesiologists or surgeons trained in anesthesiology. Nurses were involved in basic tasks including assisting during surgery and managing anesthesia equipment but were not specifically trained in anesthesia care.
2. 1990s – Early 2000s. As the healthcare system began modernizing, the need for more skilled anesthesia care providers grew, especially with the increasing number of surgeries and higher standards of patient care. Some hospitals began to train nurses in basic anesthesia procedures.
3. 2000s – Present. The Chinese government began emphasizing the development of nurse anesthetists as part of broader healthcare reforms. A series of regulations and codes were decreed to promote and govern anesthesia care space (Table). Training programs for nurse anesthetists were developed, and some universities and medical schools started offering specialized courses in anesthesia care for nursing students. This period also saw the establishment of professional organizations, such as the Chinese Society of Anesthesiology, which advocated for the formal recognition and training of nurse anesthetists. While anesthesia care has developed significantly in the past three decades, challenges remain in its continued advancement. Furthermore, the growth of anesthesia care has also led to the emergence of new obstacles.

**Table:** Anesthesia Nursing-Related Policies in China

**Policy Designation**	**Issuing Body**	**Publication Year**	**Highlights**
Notice on the Setting and Management of Outpatient Clinics and Care Units in Anesthesiology Department of Medical Institutions (National Health Commission Office Letter [2017] No. 1191)	General Office of the National Health and Family Planning Commission	2017-12-12	1. Healthcare institutions establish specialized anesthesia nursing units and enhance management protocols for outpatient anesthesia departments, involving optimizing institutional frameworks for personnel training, role allocation, professional advancement, and compensation structures. 2. The Annex outlines operational guidelines and staffing standards for anesthesia nursing units under provisional implementation.
Notice on Issuing Opinions on Strengthening and Improving Anesthesia Medical Services (National Health Commission Medical Development [2018] No. 21)	National Health Commission, National Development and Reform Commission, Ministry of Education, Ministry of Finance, Ministry of Human Resources and Social Security, National Administration of Traditional Chinese Medicine, National Healthcare Security Administration	2018-08-08	1. Nurse anesthetists shall be institutionally integrated into the organizational structure of Anesthesiology Departments within Grade 2 or higher medical facilities. 2. Nurse anesthetists are required to perform anesthesia care-related tasks under the supervision of anesthesiologists. 3. The clinical governance of anesthesia nursing services is institutionally administered through centralized coordination by the Department of Anesthesiology, establishing a systematic framework to advance specialization standards.
Notice on Guidelines for Capacity Building of Medical Services in Anesthesiology (National Health Commission Office Letter [2019] No. 884)	General Office of the National Health Commission	2019-12-09	1. Reasonable allocation of human resources according to the scope of business and clinical workload, and strengthening of the anesthesia team. 2. The ratio of nurse anesthetists to anesthesiologists in the anesthesiology (the medical care ratio in anesthesiology) specialty nursing team that cooperates with perioperative work is, in principle, not less than 0.5:1. 3. The requirements for anesthesia specialist nursing work are published in the Annex.

Note: China’s healthcare system is organized in a hierarchical structure with three main levels: Grade 3 hospitals are the highest. Grade 2 or higher medical facilities delivers routine specialty care, diagnostic and treatment equipment and performs complex surgical techniques and handles complex cases.

## Challenges in the Professional Development of Nurse Anesthetists in China

###  Insufficient Nurse Anesthetist Staffing 

 In China, as well as in many other places of the world, the volume of various surgeries has increased significantly with the advancement of medical science and technology, the demand for anesthesia care has further been amplified by the rise in endoscopic and interventional operations. The shortage of anesthesia nursing workforce has become particularly prominent. According to a 2017 national survey, there were 9147 nurse anesthetists in China, indicating a ratio of 7 nurse anesthetists for one million population.^2^ The medical care ratio in anesthesiology, which reflects the proportion of nurse anesthetists to anesthesiologists in medical institutions, is a key indicator for staffing adequacy in anesthesia care. In 2019, the Health Department of China recommended a medical care ratio in anesthesiology of no less than 0.5:1. There has been no national-level figure indicating a congregated medical care ratio in anesthesiology in China. However, recent surveys across multiple provinces reveal the medical care ratio remains below the recommended level. For instance, a 2023 survey in Guangxi province, China, found 3307 anesthesiologists and 953 nurse anesthetists in the 321 hospitals in the province, resulting in a medical care ratio of 0.3:1, up from 0.2:1 in 2017.^3^ Similarly, Gansu province and Jiangsu province reported a medical care ratio of 0.2:1 and 0.26:1, respectively, at the provincial levels.^4,5^ Due to the limited number of relevant literature retrieved and the absence of primary data collection, coupled with regional variations across provinces, potential bias may exist.

###  Lack of Standardized Training and Education System

 The development of anesthesia nursing education in China has progressed at a relatively slow pace, with a limited number of academic institutions establishing specialized anesthesia nursing programs. Consequently, clinical settings exhibit a low proportion of nurse anesthetists who have received formal higher education specifically in anesthesia care.^6^

 In 1993, Xuzhou Medical College and Nanjing Liuhe Health School in Jiang province of China, launched China’s first three-year anesthesia and emergency combined nursing program, marking the beginning of formal anesthesia care education. Over the years, Xuzhou Medical College has led the development of anesthesia nursing education programs, alongside its renowned Anesthesiology program. In 2011, Xuzhou Medical University, together with Shanghai Jiaotong University, implemented a master’s program in anesthesia care for nursing students, with a limited enrollment. In 2024, Xuzhou Medical University pioneered doctoral education in anesthesia nursing in China. As of 2024, approximately one-sixth of nurse anesthetists in China have been trained at Xuzhou Medical University, totaling more than 6000 professionals.

 Despite the leadership of Xuzhou Medical University in anesthesia nursing, formal academic education for nurse anesthetists in China is limited, with on-the-job training being the predominant method of education.^7^ Consequently, continuing training for the frontline nurse is important. The “2021 Expert Consensus on Quality Control of Anesthesiology” by the Chinese Society of Anesthesiology clearly requires training of nurse anesthetists in practice. The Chinese Nursing Association launched a training course for specialist nurse anesthetists in Beijing beginning of 2019. Over the next three years, the association successively conducted three training courses for 500 nurse anesthetists from all around China. However, training courses like this are rare and are still in exploratory phase.^8^

###  Lack of Defined Scope of Practice and Collaborative Models

 China has yet to establish a nationally unified certification system for nurse anesthetists.^9^ Most certifications are handled at the provincial level. The lack of a centralized certification mechanism has contributed to a lack of professional recognition in wide settings.^10^

 Moreover, owing to its relatively recent emergence and inadequate prioritization, China lacks clear legal regulations that define the role, scope of practice, and supervision of nurse anesthetists.^11^ Nurse anesthetists often face confusion regarding their professional responsibilities and their role within the healthcare team,^12^ leading to overlapping duties with anesthesiologists and causing disputes. Healthcare regulations explicitly state that nurse anesthetists can perform many roles under the guidelines from anesthesiologists, they should not perform anesthetic procedures including creating an artificial airway, arterial puncture, central venous puncture, intradiscal puncture, and neural blockade (referred to as the “Red Line”). Due to surgical demands and lack of anesthesiologists, nurse anesthetists are sometimes forced to perform those practices prohibited by the regulations,^4,13^ resulting in unnecessary stress.

 The primary responsibilities of nurse anesthetists generally include post-anesthetic care, pre-anesthesia preparation, post-surgical analgesia, and managing anesthesia-related supplies and equipment.^13^

 Furthermore, there are multiple models of collaboration between anesthesiologists and nurse anesthetists in China.^14^ These include the US-style model, where one anesthesiologist supervises multiple nurse anesthetists across different operating rooms. The healthcare collaborative practice model, where one anesthesiologist and one nurse anesthetist collaborate in a single operating room. Additionally, the anesthesia care coordination model assigns one anesthesiologist to each operating room, with nurse anesthetists responsible for assisting multiple rooms. A lack of national professional code for nurse anesthetists resulted in the coexistence of these different models, which may have hindered the development of a standardized approach to anesthesia care across the country.

## Strategies to Promote Nurse Anesthetist’s Development in China

###  Addressing Insufficient Nurse Anesthetist Staffing

 Financial incentives and short-term training programs can boost recruitment and retention in healthcare professions.^15^ Flexible scheduling and professional development also reduce burnout and improve job satisfaction among healthcare workers.^16^ To address the shortage of nurse anesthetists, particularly in lower-tier hospitals, the government would be to provide higher salaries, signing bonuses, housing subsidies, and tax breaks. Offering educational scholarships and flexible work schedules, especially for those with family commitments, is essential. Remote training programs using virtual classrooms and mobile simulation labs can deliver advanced education and certification to rural areas without relocation.

###  Standardizing and Expanding Education and Training

 Countries such as the US and Canada have standardized nurse anesthetist education through accredited training programs. China may establish a unified national curriculum following IFNA standards with Xuzhou Medical University as its leader.

 Additionally, establishing national and regional accreditation bodies to oversee and evaluate anesthesia nursing programs is essential. Linking accreditation to the certification process would help maintain high standards. Mandatory clinical rotations in Grade 3 hospitals may be advisable to ensure comprehensive perioperative training. Training in these high-acuity settings can provide the junior or will-be nurse anesthetists with the skills, knowledge, and confidence to manage complex cases, work in interdisciplinary teams, and deliver safe, high-quality care to surgical patients. Continuous Professional Development programs should be implemented, offering ongoing education accessible both in-person and online. These programs should focus on new anesthesia technologies, evidence-based practices, patient safety, and clinical guidelines. Continuous Professional Development should be required for certification renewal to ensure nurse anesthetists remain current with evolving best practices.

###  Developing a National Certification System and Clear Scope of Practice

 To improve anesthesia care quality and accessibility in China, a national certification authority for nurse anesthetists should be established to oversee certification, re-certification, and professional development. Nurse anesthetists should complete continuing education, similar to that for Registered Nurses in China, with around 90 hours every 5 years. This should include both theoretical and practical training, clinical competency evaluations, and specialized anesthesia education tailored to the field’s requirements. Additionally, legal frameworks must be created to protect nurse anesthetists’ professional rights and ensure liability coverage. Evidence from countries like the US, UK, and Canada demonstrates that a structured certification system, clear scope of practice, and regional adaptations can improve patient outcomes and workforce competency, particularly in underserved areas.

 To prevent nurse anesthetists from crossing the “Red Line” of prohibited procedures, clear role definitions must be established, and supported by targeted education and training. Nurse anesthetists should always work under anesthesiologists’ supervision for high-risk tasks, and standardized protocols should define permissible procedures. Implementing accountability systems with regular audits will ensure adherence to these guidelines and prevent violations of scope, ultimately safeguarding the integrity of anesthesia care.

###  Optimizing Collaborative Models for Nurse Anesthetists in China

 To enhance collaborative models for nurse anesthetists in China, it is crucial to establish clear guidelines that define their roles within anesthesia teams. Joint training programs for nurse anesthetists and anesthesiologists can significantly improve communication, coordination, and teamwork, as demonstrated in countries such as the US and UK, where such initiatives have led to better patient outcomes and a reduction in errors.^17^ These programs emphasize shared clinical competencies and a mutual understanding of each other’s professional roles.

 The management of nurse anesthetists in Chinese hospitals is currently inconsistent, with some nurse anesthetists being managed under anesthesiology, others under the operating room, or both. This fragmentation can create conflicting expectations and impede collaboration. Evidence from Canada indicates clearly defined roles within teams enhance efficiency and safety.^18,19^ Therefore, China should implement a unified management system for nurse anesthetists, ensuring consistent policies, clear reporting lines, and fostering professional development. This approach would improve team dynamics, enhance the quality of care, and support the professional growth of nurse anesthetists.

###  Advocacy and Policy Reform

 To advance advocacy and policy reform for nurse anesthetists in China, it is essential to focus on lobbying for their formal recognition and expanded roles. Strengthening advocacy through collaboration with anesthesiologists and healthcare groups is critical, while evidence-based proposals demonstrating nurse anesthetists’ positive impact on patient outcomes will support policy change. Public awareness campaigns and ongoing dialogue with policy-makers are vital for long-term success. Research from countries such as Canada shows that educational initiatives increase recognition,^20^ while continuous engagement with policy-makers ensures that nurse anesthetists’ evolving needs are addressed. Promoting research on the impact of nurse anesthetists will facilitate further integration of nurse anesthetists into China’s healthcare system.

## Conclusions

 As an emerging specialty, anesthesia care in China faces multifaceted challenges, including educational gaps, regulatory voids, and role ambiguity. Addressing these issues requires tailored strategies that integrate global best practices with China’s unique healthcare context. By prioritizing education reform, policy development, and interdisciplinary collaboration, China can cultivate a robust nurse anesthetist workforce to meet evolving clinical demands and enhance perioperative care quality.

## Disclosure of artificial intelligence (AI) use

 Not applicable.

## Ethical issues

 Not applicable.

## Conflicts of interest

 Authors declare that they have no conflicts of interest.

## Disclaimer

 The views expressed in this article are views of the authors and do not represent views of the organisation they work for.
